# Bioinformatics Prediction of SARS-CoV-2 Epitopes as Vaccine Candidates for the Colombian Population

**DOI:** 10.3390/vaccines9070797

**Published:** 2021-07-17

**Authors:** Diana Montes-Grajales, Jesus Olivero-Verbel

**Affiliations:** Environmental and Computational Chemistry Group, School of Pharmaceutical Sciences, Zaragocilla Campus, University of Cartagena, Cartagena 130015, Colombia; dmontesg@unicartagena.edu.co

**Keywords:** severe acute respiratory syndrome, immuno-informatics, HLA, vaccine design, T-cell epitope, peptide, interaction, immunogenicity

## Abstract

Coronavirus disease (COVID-19) pandemic caused by the coronavirus SARS-CoV-2 represents an enormous challenge to global public health, with thousands of infections and deaths in over 200 countries worldwide. The purpose of this study was to identify SARS-CoV-2 epitopes with potential to interact in silico with the alleles of the human leukocyte antigen class I (HLA I) and class II (HLA II) commonly found in the Colombian population to promote both CD4 and CD8 immune responses against this virus. The generation and evaluation of the peptides in terms of HLA I and HLA II binding, immune response, toxicity and allergenicity were performed by using computer-aided tools, such as NetMHCpan 4.1, NetMHCIIpan 4.0, VaxiJem, ToxinPred and AllerTop. Furthermore, the interaction between the predicted epitopes with HLA I and HLA II proteins frequently found in the Colombian population was studied through molecular docking simulations in AutoDock Vina and interaction analysis in LigPlot+. One of the promising peptides proposed in this study is the HLA I epitope YQPYRVVVL, which displayed an estimated coverage of over 82% and 96% for the Colombian and worldwide population, respectively. These findings could be useful for the design of new epitope-vaccines that include Colombia among their population target.

## 1. Introduction

Coronavirus disease (COVID-19), was declared a global pandemic by the World Health Organization on 11 March 2020. This infection has affected more than 200 countries [1], with over 183 million cases and 3971,687 deaths worldwide by 6th July, 2021 [2]. At this time, the Region of Americas continues to account for around 50% of all deaths and 40% of all cases worldwide [2], Colombia being the 11th country with the highest number of cumulative cases and deaths [3]. This disease caused by the coronavirus SARS-CoV-2 exhibits a wide range of manifestations from non-symptomatic and mild illness (mainly associated with cough, fever, fatigue, sore throat, headache and muscle pain) to pneumonia and acute respiratory distress syndrome [4]. This is characterized by lung collapse, the requirement of ventilatory assistance and oxygen support, and has been related to multi-organ collapse and hyperinflammatory states in extremely severe cases [5,6]. The latter is mediated by a cytokine storm, which could be induced by the nucleocapsid protein (N), and to a lesser extent by the spike protein (S) of SARS-CoV-2 [7].

The long-term immunity of the vaccines and their effectiveness against reinfections of SARS-CoV-2 is still uncertain [8]. Some authors suggested that the virus is likely to continue present in the population [9]. Therefore, the improvement of vaccine development and production capacities in several countries and continents is essential. Especially in Latin America, which is one of the most affected areas by COVID-19 pandemics.

SARS-CoV-2 belongs to the beta genus of the *Coronaviridae* family, a group of single stranded positive sense RNA viruses able to affect humans and animals [10]. The name of this type of viruses comes from the Latin word “corona” that means crown, given due to the appearance of the lipid envelope of the virions, which presents distinctive club-shaped projections [11]. Its viral genome ranges approximately from 27 to 32 kilobases in size, and encodes: the spike (S), membrane (M), envelope (E) and nucleocapsid (N) proteins [12], which are the structural proteins of the virus, as well as 16 non-structural proteins (NSP1 to NSP16), accessory protein chains [5], the main protease (also known as 3C-like proteinase; 3CLpro), and the papain-like protease (PLpro) [13].

The S protein mediates the viral entry through its interaction with the human angiotensin-converting enzyme 2 (ACE 2), which is its functional receptor [14]. The E protein is involved in virus pathogenicity, it has been found to participate the release of the viruses and the activation of the inflammasome [15]. The N protein has been related to multiple processes, including the packaging of the viral genome, the viral RNA-protein (vRNP) assembly through its interaction with the M protein, as well as the promotion of RNA template switching and the recruit of host factors to promote RNA synthesis [16]. The M protein cooperates with the other structural proteins of SARS-CoV-2. It has been found to stabilize the N protein during the vRNP assembly and to support the S protein in the attachment to the host cells and viral entry [17].

Several authors have reported epitope-based vaccine candidates by using inmmunoinformatic approaches [18]. These have predicted peptides with potential to interact with Human Leukocyte Antigen class I (HLA I) and class II (HLA II), and exhibited immunogenic responses, non-toxicity and non-allergenicity [19]. These are based on the identification of T-cell or B-Cell epitopes in the SARS-CoV-2 proteome, especially focused on the S protein [20] or the structural proteins (S, E, M, and N) of this virus [21]. Some of them, such as the S [22], and N [23,24] proteins have been shown to be immunogenic [25]. Adaptive immunity, mediated by lymphocytes, could be generated by most of the vaccines. In order to neutralize the virus, B lymphocytes produce antibodies through the participation of CD4 T cells, also called helper T cells. Similarly, infected cells could be directly destroyed by CD8 T cells, known as cytotoxic T cells [26]. The activation of CD8 T cells and CD4 T cells is mediated by the HLA I and HLA II, respectively. These HLAs are involved in antigen presentation to T cells. The rationale of epitope-based vaccines is to identify peptides able to bind strongly to HLAs and elicit immunogenicity through the activation of T cells [27] by preparing the body to fight against SARS-CoV-2 infection [19]. 

In summary, T-cells epitopes may elicit cytotoxic or/and immunogenic responses against SARS-CoV-2 through the activation of CD4 T-cell receptor (TCR) and CD8 TCR, which are mediated by the peptide binding to HLA I and HLA II, respectively. Therefore, the aim of this research was to identify epitopes in structural proteins of SARS-CoV-2 based on HLA I and HLA II commonly present in the Colombian population. The promising epitopes may have applications in the design of peptide-based vaccines and the development of diagnostic tests. In addition, a systematic review was performed to find HLA I and HLA II alleles commonly found in the Colombian population, which helped to determine the estimated coverage of the promising peptides in the country and complement the repositories of HLAs reported for Latin America [21], with the inclusion of more datasets from scientific articles.

## 2. Materials and Methods

### 2.1. Materials

All in silico predictions and data analysis were carried out using a Dell Precision 3630 Tower (Dell, Beijing, China) workstation equipped with Intel Core i7-9700K CPU at 3.60 GHz (8 cores), 64 GB RAM, and GPU (NVIDIA Quadro P620 with 2 GB memory). The operating system utilized were Windows 7 Professional and Ubuntu 18.04.5 LTS (Linux), running on Oracle Virtual Machine. 

### 2.2. Literature Search of HLAs Frequencies

In order to identify HLAs (HLA I and HLA II) commonly found in the Colombian population, a literature search was performed on PubMed (https://pubmed.ncbi.nlm.nih.gov/, accessed on 24 February 2021), Web of Science (http://www.webofknowledge.com/, accessed on 24 February 2021), and Science Direct (https://www.sciencedirect.com/, accessed on 24 February 2021). A single query was utilized to search for articles reporting HLA I frequencies in the Colombian population (Table 1). On the other hand, HLA II genes reported in the IPD-IMGT/HLA database (http://www.ebi.ac.uk/ipd/imgt/hla/, accessed on 7 February 2021) were considered for the identification HLA II allelic frequencies in Colombia. The name of each gene was used to perform a preliminary search on PubMed, by using the generic query: “Name of the HLA Class II gene” AND “Colombia” (Eg. “DRA” and Colombia”). Accordingly, the genes: DRA, DQA2, DPA2, DPB2, DMA, DMB, DOA, DOB, DRB2, DRB6, DRB7, DRB8 and DRB9 did not present any results about their frequency in the Colombian population. Therefore, these were not included in the final queries to select articles reporting the frequencies of HLA II alleles. The literature search for this type of alleles were divided in three queries as the maximum number of Boolean connectors (AND/OR) allowed in Science Direct was eight (Table 1). 

Research and review articles, as well as short/brief communications in English and Spanish were considered. The outcomes for HLA I and HLA II alleles were processed separately. The results obtained in PubMed, Web of Science and Science Direct for the corresponding queries were downloaded in the reference formats: BibTex (Web of Science and Science Direct) and nbib (PubMed). Subsequently, two folders (HLA I, and HLA II) were created in the reference manager Mendeley Desktop v 1.19.4, and the reference files uploaded accordingly. The tool “Check for duplicates” of this software was used to identify and delete redundant articles. 

The information contained in the title and abstract was used to perform a first screening and to remove the articles that did not present data about the prevalence of HLA I and HLA II alleles in the Colombian population. In addition, the reports of allelic frequencies in groups with associated diseases (Eg. lupus, arthritis, diabetes, hepatitis, autoimmune diseases, multiple sclerosis) were not considered for further analysis. 

On the other hand, additional datasets were retrieved from the Allele frequency net database (AFND) [28], and included in this study. The HLA searching option used in this database was “HLA classical allele freq search” with the following parameters, country: Colombia, population standard: gold and silver, sort by: Allele (highest to lowest frequency), and level of resolution: two field. The options: population, source of the dataset, ethnic origin, region, type of the study, sample year, sample size, and frequencies were configured to show all the possible results. 

The selected articles from PubMed, Web of Science, Science Direct and AFND were downloaded in pdf format, carefully reviewed, and used to generate an excel file with HLA I and HLA II allelic frequencies reported in Colombia. The cumulative frequency of each dataset was calculated through a customized python script (Python_Script_CumFreq.py, available in the Supplementary Materials), and only articles reporting allelic frequencies whose sum was 100 ± 2% were kept, except for DRB2-DRB5, as these genes are not expected to be present in all individuals. Furthermore, alleles with low resolution were removed (less than two field resolution). The number of results obtained of all the searches were last updated by 24 February 2021, and the process was documented through a PRISMA flow diagram. The HLA allelic frequencies obtained through the systematic search were sorted in descending order. 

### 2.3. HLAs Selection for Epitope Prediction

The dataset of HLA I alleles used for epitope prediction included: (1) HLA-A, HLA-B and HLA-C alleles that presented the top ten highest frequencies in the Colombian general population (Dataset: Colombia-Bogotá). As well as, (2) the HLA-A, HLA-B and HLA-C alleles that exhibited the highest frequency for each of the eleven Colombian Amerindian groups with HLA I frequencies reported in the AFND [28], which coincided with the results of the literature search [29,30,31]. These Amerindian groups were: Arhuaco, Embera, Inga, Kogi, Chimila Norte, Wiwa Norte, Waunana, Wayyu, Zenú, Ticuna Arara, and Ticuna Tarapaca. Weighted allele frequencies (WAFs) were not calculated for HLA I alleles in the Colombian population as each dataset came from a single article [32]. Unfortunately, no reports about the frequency of HLA I alleles in African Colombians were found.

Due to the extensive amount of data, a python script was developed to obtain the WAFs by calculating the weighted average of HLA II allele frequencies for the Colombian population grouped by ethnicity (Mestizos, African Colombians and Colombian Amerindians); and each of the reported alleles were expressed in two-field format (Python_Script_WAF.py, available in the Supplementary Materials). These scripts were used to generate an excel table containing the WAFs and number of individuals with a specific HLA II allele in each of the three ethnic groups considered, as well as the reported frequencies for HLA I alleles. All the HLA II alleles exhibiting more than 5% in at least one of the studied ethnic groups were selected for epitope prediction and in silico evaluation. These were also used to perform a Venn diagram (http://bioinformatics.psb.ugent.be, accessed on 16 July 2021) in order to distinguish HLA II alleles with high frequencies in several ethnic groups.

### 2.4. T-Cell Epitope Prediction

The prediction of CD4 and CD8 T-cell epitopes was conducted by using the NCBI reference sequence of non-structural proteins SARS-CoV-2 (Table 2). In addition to the selected HLA I and HLA II alleles commonly found in the Colombian population.

HLA I epitope predictions were performed on NetMHCpan 4.1 [33] by using the following parameters, peptide length: 8–12, threshold for strong binder: 0.5% rank, threshold for weak binder: 2% rank, inclusion of theoretical binding affinity (predicted IC_50_ values). Short peptides (8–12 amino acids) were generated and evaluated to identify candidate epitopes with high affinity for the alleles HLA-A, HLA-B and HLA-C commonly found in the Colombian population in this server. On the other hand, HLA II epitope predictions were based on DRB1 alleles highly frequent in the Colombian population and performed on a NetMHCIIpan 4.0 server [33]. The parameters employed were peptide length of 15 amino acids, threshold for strong binder of 1% rank, threshold for weak binder of 5% rank, and inclusion of the binding affinity predictions.

Data analysis was performed in Python 3 through customized scripts. The function of these scripts were to group the epitopes predicted by NetMHCpan 4.1 [33] and NetMHCIIpan 4.0 [33] servers as strong or weak binders, and to retrieve the names and number of the interacting alleles per peptide. Both NetMHCpan 4.1 [33] and NetMHCIIpan 4.0 [33] reported if the sequence of each peptide was a strong binder (SB) or a weak binder (WB) with each of the HLAs included in the analysis [33]. These data were retrieved in a column called “bind level” in the result table generated by these servers. The scripts developed in this research used the resultant tables as input files to count the number of HLAs interacting as SB or WB with each peptide by using the “groupby()” function to group the data by both “peptide” and “bind level” at the same time. Subsequently, the scripts counted the number of HLAs in each group [HLAs with the same peptide and bind level (SB or WB)] with the “count()” function to retrieve the number and names of the HLAs interacting with each peptide as SB or WB. The top ten peptides with the highest number of interacting alleles with strong affinity were kept for further analysis (Analysis_HLA_I.py, Analysis_HLA_II.py, and Interactions_Summary.py). These are available in the Supplementary Materials.

In addition, the coverage of the promising epitopes for the worldwide population was predicted by using the Population Coverage Calculation Tool of IEDB (http://tools.iedb.org/population/, accessed on 31 March 2021), with the following parameters: Class I and Class II combined and area: world. The information of the MHC restricted epitopes was completed with the HLAs predicted to interact with these peptides (strong or weak binders) by NetMHCpan 4.1 [33] and NetMHCIIpan 4.0 [33].

Each promising peptide was predicted to interact with several HLAs. Therefore, a Coverage Score (CS) was defined to a calculated single value representing the coverage in the Colombian general population in the case of HLA I alleles, and the coverage per each ethnic group in the case of HLA II. Information regarding allelic frequencies for HLA I was very scarce (each dataset came from a single article), therefore WAF were not calculated and the estimated coverage was defined for HLA-A, HLA-B and HLA-C as the cumulative frequencies (sum of the frequencies) of the alleles interacting as strong or weak binders with each peptide, in Colombian general population. On the other hand, the estimated coverage for HLA II (DRB1) was calculated as the WAF of the interacting alleles per peptide. The calculated coverage scores were useful to selected candidate peptides with the highest estimated coverages. However, the low number of articles reporting HLAs frequencies for the Colombian population is a limitation and may affect the accuracy of the estimates. 

The customized scripts developed to calculate estimated coverages used the “groupby()” function to group the data according to the peptide sequence, bind level and type of HLA (HLA-A, HLA-B, HLA-C and HLA-DRB1; other HLA II alleles were not considered as they are not expected to be present in all individuals),as well as, the sum the frequencies of the interacting alleles for HLA I and the WAF for HLA II.

Promising epitopes were submitted to Vaxijen v. 2.0 [34] to assess their immunogenicity in silico, by selecting viruses as target entities and default threshold. In addition, Allertop 2.0 [35] was used to predict allergenicity, and Toxinpred [36] was utilized to evaluate the theoretical toxicity with default parameters. Among these, SVM (Swissprot) based method, E-value cut-off for motif-based method of 10, SVM threshold of 0, and calculation of the following physicochemical properties: hydrophobicity, charge and molecular weight. In addition, the potential of the promising epitopes to induce the release of TNF gamma was evaluated in silico by using IFNepitope server (http://crdd.osdd.net/raghava/ifnepitope/, accessed on 31 March 2021).

### 2.5. Peptide-Protein Docking Studies

Theoretical binding affinities were calculated by molecular docking simulations to determine the possible interaction between the promising peptides and HLAs commonly found in the Colombian population. In order to do that, a blind docking strategy was used in AutoDock Vina, this software calculates in silico binding affinities and retrieves information regarding the predicted pose and binding pocket of the peptides with the highest (absolute value) affinity scores. The structures of the promising epitopes were previously generated by modelling on Pep-Fold 3.0 server [37]. On the other hand, HLA I and HLA II selected to be highly frequent in the Colombian population with three-dimensional structures available in Protein Data Bank (PDB) [38] were downloaded in pdb format. The names of the proteins and their PDB identifiers (PDB ID) are available in Table S1. Subsequently, all ions, water molecules and other substructures were removed and the protein structures were prepared by using the biopolymer structure preparation tool of the in Sybyl X-2.0 (Tripos, St. Lous, MO, USA) with default settings. The resultant coordinates were optimized in the same software with the following parameters: Powell method, Kollman United and Kollman All Atoms force fields, AMBER charges, dielectric constant of 1.0, nonbonded (NB) cutoff of 8.0, maximum interactions of 100 and termination gradient of 0.001 kcal/mol. Finally, the size and coordinates of the center of the grid containing the whole protein structure were determined, by using a spacing of 0.375 Å, and the resultant structures saved as pdbqt in AutoDock Tools (MGL Tools) [39]. These parameters and files were used as input for docking in AutoDock Vina [40], along with the following settings: twenty number of modes, energy range of 1.5, and exhaustiveness of 25. The predicted docking affinity scores were ranked and used to identify the peptide-protein complexes with the highest (absolute value) affinity scores. In order to better visualize these results, a heatmap with clustering trees was generated with the heatmap.2 function of the statistical program R version 3.6.3. [41,42]. 

### 2.6. Interactions Analysis and Molecular Dynamics

The epitopes with the highest (absolute values) affinity scores predicted by AutoDock Vina were submitted to interaction analysis using LigPlot+ [43]. This program was utilized with default parameters. In addition, a short molecular dynamics (MD) simulation was performed to further study the interaction of the HLA-peptide complex containing the promising epitope obtained from the receptor-binding domain of the S protein of SARS-CoV-2 that presented the highest (absolute value) affinity score in silico. The MD was carried out in Gromacs (version 2020.2) [44], by using the Chemistry at Harvard Macromolecular Mechanics (CHARMM) force field [45]. The peptide-protein complex was solvated by placing it into the center of a cubic box filled with water, 1.0 nm from the boundaries of the complex. After that, ions were added to neutralize the system, followed by a constant pressure (NVT) equilibrium simulation for 1 ns with a time step of 2 fs and reference temperature of 300 K. A second equilibrium step was carried out for 1 ns by using a constant particle number, pressure, and temperature (NPT) ensemble. The production step of the MD simulation was executed during 10 ns under isothermal–isobaric conditions, with time step: 2 fs, reference temperature: 300 K, pressure 1 bar, van der Waals cutoff: 1.2 nm, and grid spacing: 0.16 nm using the leap-frog integrator and Verlet cutoff scheme. The atomic coordinates were recorded every 10 ps to obtain 1000 different molecular conformations. The same procedure was carried out with the peptide-free protein (HLA) for comparative purposes [46], by measuring the root-mean square deviations (RMSD). In addition, the root-mean square fluctuations (RMSF) of the residues of HLA-B*08:01 (backbone) were computed using the trajectories of the MD simulation.

## 3. Results

### 3.1. Literature Search of HLAs Frequencies

The literature search for HLA I frequencies in the Colombian population retrieved 486 articles. These were obtained from PubMed, Web of Science and Science Direct by using the following query: (“MHC Class I” OR “MHC I” OR “HLA Class I” OR “HLA I” OR “HLA-A” OR “HLA-B” OR “HLA-C”) AND “Colombia” (Table 3). On the other hand, the systematic search for HLA II frequencies in the Colombian population retrieved 1057 articles (Table 4). This search was carried out through the combination of three different queries in PubMed, Web of Science and Science Direct. Furthermore, a total of 12 and 39 datasets referring HLA I and HLA II frequencies in Colombia were retrieved from AFND [28], respectively.

**Figure 1:**
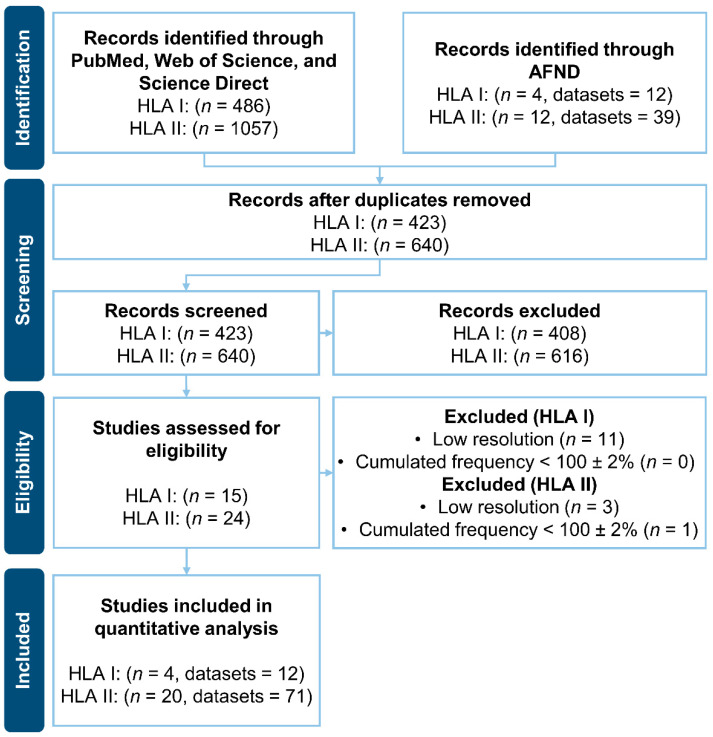
PRISMA flow diagram of the systematic literature search of the frequencies of the alleles HLA class I (HLA I) and HLA class II (HLA II) in the the Colombian population by using PubMed, Web of Science and Science Direct, as well as the Allele Frequency Net Database (AFND). The number of records (*n*) represent the number of articles, which may contain multiple datasets. Each dataset is defined by a unique combination of: population and reference as usually presented in the grid format of AFND.

After duplicates removal, and manual screening of the titles and abstracts, 15 and 24 articles were accessed for eligibility in the groups of HLA I and HLA II alleles, respectively. Only articles reporting HLAs allelic frequencies with two-field resolution and cumulated frequencies of 100 ± 2% were maintained. A PRISMA flow diagram showing the data collection process is presented in Figure 1.

### 3.2. HLAs Selection for Epitope Prediction

#### 3.2.1. HLA I

The total set of HLA I allelic frequencies reported for the Colombian population, including Colombian Amerindian groups, according to the systematic search are presented in Table S2. The HLA I allelic frequencies in the general Colombian population (Dataset: Colombia-Bogotá) obtained from AFND [28] were used to identify the 10 most frequent HLA-A, HLA-B and HLA-C alleles (Table 5). Similarly, the data corresponding to the Native American groups: Arhuaco, Embera, Inga, Kogi, Chimila Norte, Wiwa Norte, Waunana, Wayyu, Zenú, Ticuna Arara and Ticuna Tarapaca were used to identify the most frequent HLA-A, HLA-B and HLA-C alleles in each of these populations (Table 6). The sum of the top-10 frequencies for the HLA-A, HLA-B and HLA-C alleles in the general Colombian population (Group: Colombia Bogotá) was 0.736, 0.523 and 0.778, respectively. All Colombian Native American groups showed HLA-A*24: 02 as the most frequent HLA-A allele, which is also the most common for the general population (Dataset: Colombia-Bogotá). On the other hand, the most frequent HLA-B and HLA-C alleles reported for Colombian Amerindian groups showed a greater variability.

#### 3.2.2. HLA II

The complete set of HLA II allelic frequencies reported for the Colombian population, including Colombian Amerindian groups and African Colombians, are presented in Table S3. Furthermore, WAFs of HLA II in the Colombian population were calculated as the weighted average of the frequencies obtained from the literature search and AFND [28]. This information grouped by ethnicity and the specific alleles in two-field formats are presented in Table S4. Alleles with WAFs > 5% in each ethnic group (Mestizo, African American and Colombian Amerinds) were selected for further analysis (Table 7).

**Figure 2:**
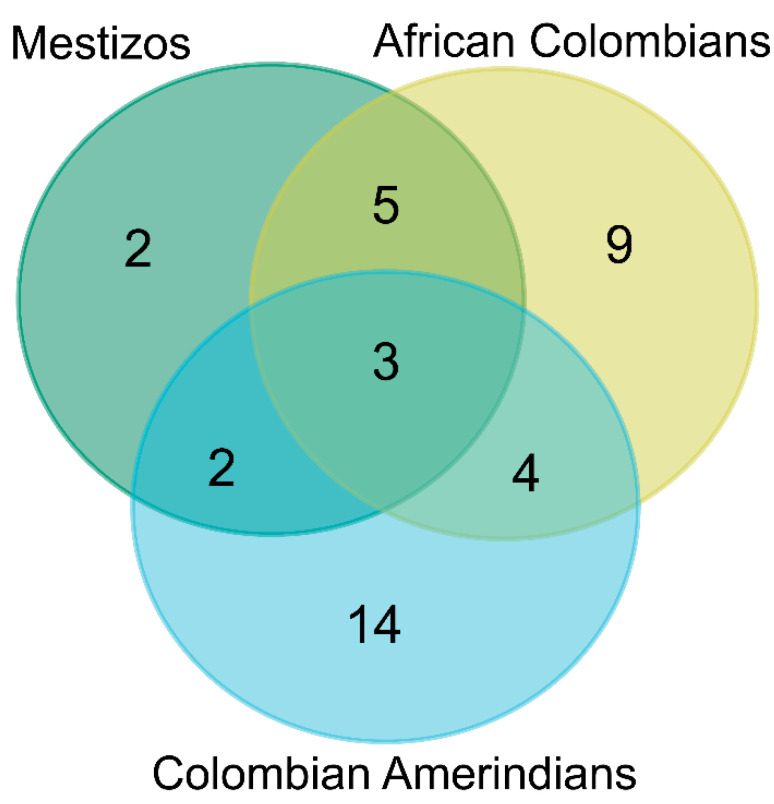
Venn diagram of the HLA II alleles with weighted allele frequencies (WAFs) over 5% among ethnicities reported in the Colombian population.

According to the Venn diagram (Figure 2), only three HLA II alleles exhibited over 5% of WAFs in the three main ethnic categories of the Colombian population were considered in this study (Mestizo, African Colombian, and Colombian Amerindians). These were DQB1*03:02, DQB1*03:01, and DQB1*04:02. Mestizos and African Colombians who share five high frequency alleles (WAFs > 5%): DQB1*06:02, DRB1*07:01, DQB1*05:01, DQB1*02:01, and DRB1*03:01. Mestizos and Colombian Amerindians presented two common alleles with WAFs > 5%: DRB1*08:02 and DRB1*04:07. Besides, African Colombians and Colombian Amerindians share the HLA II alleles DQA1*05:01, DRB1*08:04, DQA1*04:01, DQA1*03:01 with WAFs > 5% in each group. These alleles are proposed as key targets in the development of HLA II epitopes focused to cover different ethnicities of Colombia. 

### 3.3. T-Cell Epitope Prediction

T cell epitopes were generated based on 34 HLA I and 19 HLA II alleles commonly found in the Colombian population and available in the servers used for epitope prediction (Table 8). These were the top-ten HLA-A, HLA-B and HLA-C most frequent alleles per type found in the Colombian general population (Dataset: Colombia-Bogotá) and the most common HLA-A, HLA-B and HLA-C alleles found in each of the Colombian Amerindian groups with reports of HLA I. As well as, HLA II alleles reported to be present with high frequency (WAFs > 5%) in the Colombian population.

HLA I epitopes were generated based on the SARS-CoV-2 proteins S (Table S5), N (Table S6), E (Table S7) and M (Table S8). Similarly, HLA II epitopes were generated from each of these viral structural proteins (Tables S9–S12). The promising peptides (with the highest number of strong interactions for HLA-I and HLA-II commonly found in the Colombian population) that exhibited predicted immunogenicity, non-toxicity and non-allergenicity are shown in Table 9. 

According to the immunoinformatics analysis of these peptides (Table 9), only four promising epitopes were predicted to induce the release of TNF gamma by the TNFepitope server (Table 10). The coverage calculation performed on IEDB for these epitopes showed that they are predicted to cover up to 96.62% of the worldwide population. 

These four promising epitopes are located in the S and N proteins of SARS-CoV-2. The peptides YQPYRVVVL and RAAEIRASANLAATK are placed in the receptor-binding domain (RBD) and the central helix (CH) of the S protein, respectively. On the other hand, SPDDQIGYY and QFAPSASAF are positioned in the N-terminal domain (NTD) and the C-terminal domain (CTD) of the N protein of SARS-CoV-2, respectively.

### 3.4. Peptide-Protein Docking Studies

**Figure 3:**
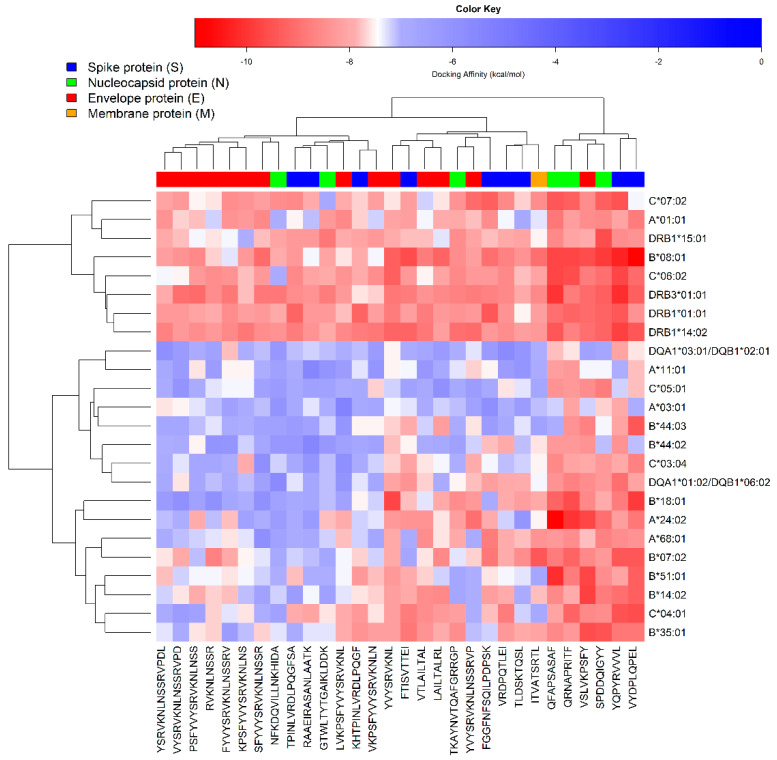
Heatmap representing the calculated docking affinity values (kcal/mol) of HLAs interacting with promising epitopes that exhibited immunogenicity, non-toxicity and non-allergenicity in silico.

Confirmatory peptide-protein docking studies were carried out with AutoDock Vina [40,47]. The predicted binding affinity scores (kcal/mol) of HLAs interacting with promising peptides that showed immunogenicity, non-toxicity and non-allergenicity in silico are presented in Table S13, and represented as a heatmap with dendrograms in Figure 3. All the studied peptides exhibited high (absolute value) affinity scores with at least one of the evaluated alleles. Besides, three of the promising peptides that were predicted to induce the release of TNF gamma (YQPYRVVVL, QFAPSASAF and SPDDQIGYY) showed a multi-target behavior, by interacting with most of the HLAs used for docking studies. These were used for further analysis along with the other peptide that was predicted to induce the release of TNF gamma in silico (RAAEIRASANLAATK). 

### 3.5. Interactions Analysis and Molecular Dynamics

**Figure 4:**
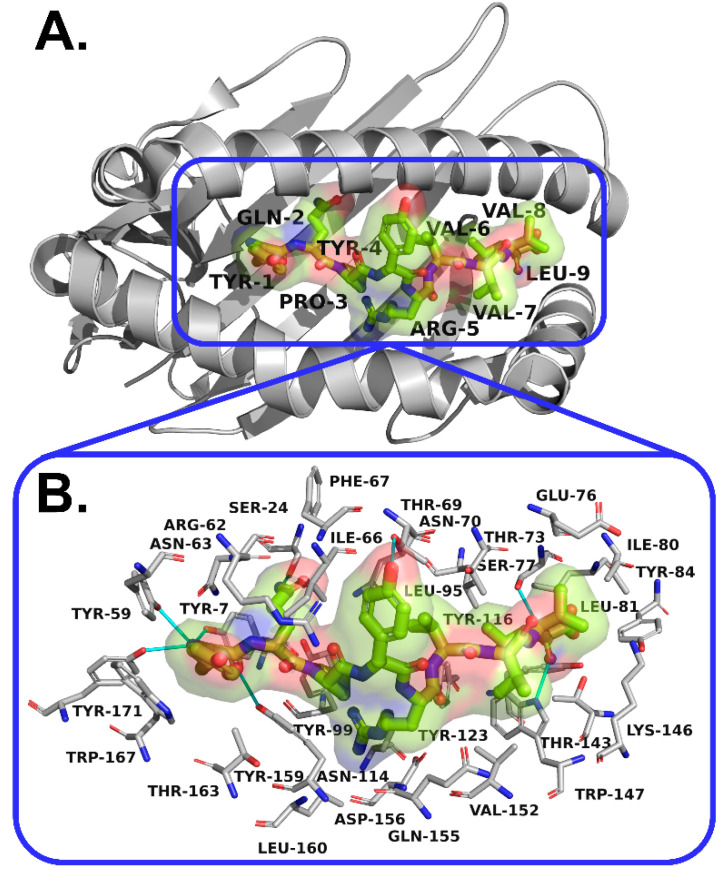
(**A**) Three-dimensional view of the complex formed by the peptide YQPYRVVVL with HLA-B*08:01 (PDB: 3X13); showing (**B**) the binding site and interactions predicted by LigPlot+. Hydrogen bonds are represented with lines in cyan.

The interaction analysis between the promising peptides predicted to induce the release of TNF gamma and four of the most common HLAs in the Colombian population was carried out by using LigPlot+ [43]. The three-dimensional view of the complexes and the interactions between these peptides with HLA-A*24:02, HLA-B*51:04, HLA-C*04:01, and HLA-DQB1*06:02 are presented in Figures S1–S4, respectively. All the promising epitopes were predicted to interact with the peptide-binding cleft of these HLAs. In addition, the three-dimensional view of the complex formed by the promising epitope with the greatest estimated coverage (YQPYRVVVL) and the protein (HLA-B*08:01) that exhibited the highest (absolute value) affinity score with this peptide (−10.3 kcal/mol) is presented in Figure 4.

**Figure 5:**
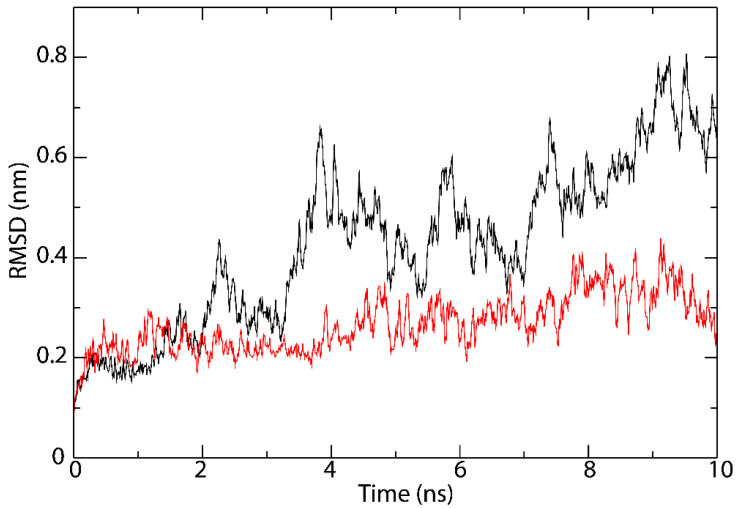
Molecular dynamics (MD) simulation of the peptide/protein complex between YQPYRVVVL and HLA-B*08:01 (black), and the peptide-free protein HLA-B*08:01 (red).

The MD simulation (Figure 5) confirmed the peptide induced conformational change that has been reported for the binding of epitopes with HLA I [48] and HLA II [46] proteins. The average RMSD of the atomic positions for the dynamics and static models of the protein-peptide complex and the peptide-free protein were 4.27 Å and 2.67 Å, respectively. 

**Figure 6:**
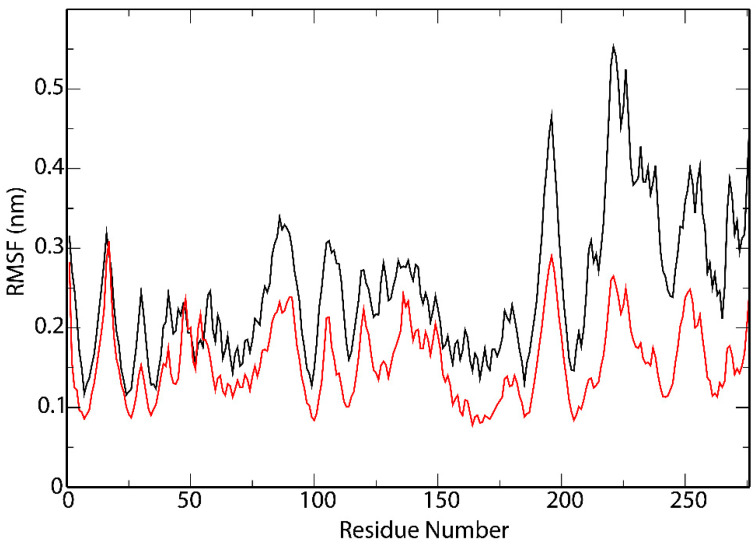
Root-mean square fluctuations (RMSF) values of the backbone through the Molecular dynamics (MD) simulation of the peptide/protein complex between YQPYRVVVL and HLA-B*08:01 (black), and the peptide-free protein HLA-B*08:01 (red).

The RMSF analysis (Figure 6) revealed the flexibility of HLA-B*08:01. The binding of the epitope YQPYRVVVL resulted in a similar fluctuation pattern with notorious differences in the RMSF values near the residues: ASP30, GLU58-ALA90, GLY104-ARG181, PRO193-GLU198 and ALA211-PRO276, which indicates that the binding to this epitope may influence conformational changes around these amino acids.

## 4. Discussion

Immunoinformatics has been used for the prediction of epitopes of SARS-CoV2 [19,49,50], as T-cells may be crucial to combat this virus causing COVID-19 [19]. The state of art regardissssng HLAs frequencies in Latin America is very limited [21], which is concerning as this is one of the most affected areas for the pandemics. In this article, we performed a systematic review to find HLAs (HLA I and HLA II) allelic frequencies reported for the Colombian population. This expanded the number of organized datasets reporting HLAs allelic frequencies for the Colombian population from seven [21] to twelve for HLA I and seventy one for HLA II.

The design of novel vaccines or treatments against COVID-19 are needed to cover the worldwide demand, especially in developing countries as Colombia. A computational approach was used to predict SARS-CoV-2 epitopes, as this approach has been shown to speed up the screening process of peptide libraries [47]. Hereby, we report four promising epitopes that presented immunogenicity, non-toxicity, non-allergenicity and potential to release TNF-gamma in silico. These are YQPYRVVVL and RAAEIRASANLAATK, which are based on the S protein of SARS-CoV-2, as well as, QFAPSASAF and SPDDQIGYY which are based on the N protein of this virus. Both structural proteins, N and S, have been reported to present immunogenic activity.

The promising epitopes based on the S protein of SARS-CoV-2 (YQPYRVVVL and RAAEIRASANLAATK) proposed herein are conserved in the current variants of concern: Alpha (United Kingdom), Beta (South Africa), Gamma (Brazil) and Delta (India); as well as in all variants of interest: Eta (Multiple countries), Iota (United States of America), Kappa (India) and Lambda (Peru) [51]. The prioritization of epitopes like these that are conserved across variants of concern and interest of SARS-CoV-2 is crucial to prevent immune evasion due to viral genomic diversity [52].

The promising epitope YQPYRVVVL has been reported to exhibit high antigenicity against the beta variant from South Africa (GSAID ID: EPI_ISL_1706561) and another variant from India (GSAID ID: EPI_ISL_1708422) [53]. Furthermore, this peptide exhibited high binding affinity for several HLAs in silico and has been proposed as candidate epitope for vaccine design [54]. On the other hand, the promising HLA II epitope RAAEIRASANLAATK has been reported to exhibit a good coverage in other Latin American countries, including Argentina, Bolivia, Brazil, Chile, Ecuador, Paraguay, Peru and Venezuela [21]. Furthermore, the candidate epitopes based on the N protein of SARS-CoV-2 QFAPSASAF and SPDDQIGYY have been described as promising epitopes for the development of multi-epitope vaccines [55]. Therefore, the promising peptides described herein are not only restricted to the Colombian population, but also can be useful for the development of peptide-based vaccines for several countries.

According to the IEDB, the promising epitopes proposed in this article can exhibit up to 96.62% of coverage in worldwide population. In addition, the estimated coverage of the peptide VYDPLQPEL calculated for the Colombian population based on allelic frequencies indicated this could cover up to 82.07% of the population through its binding with HLA-C proteins, and present a coverage of 50.85% and 34.39% associated to its interaction with HLA-A and HLA-B in the Colombian population.

The structural analysis carried out with AutoDock Vina [40] and LigPlot+ [43] showed that the promising peptides interacted with the expected binding site of the studied HLAs, in the peptide-binding groove [56]. Most of the interactions where hydrophobic with the presence of some hydrogen bonds. In addition, the contact residues predicted for YQPYRVVVL with HLA-B*08:01 revealed the interaction of this promising epitope with two amino acids in the positions 156 and 116 that have been reported as crucial for peptide recognition of HLAs [56], ASP156 and TYR116.

The MD suggested a conformational change induced by the peptide binding, in the complex formed by the epitope with the highest coverage and the protein that presented the highest (absolute) value affinity score for it (VYDPLQPEL/HLA-B*08:01). This is in agreement with previous reports for similar systems of HLA proteins [46,48]. In addition, the RMSF pattern presented for the binding of the promising SARS-CoV-2 epitope VYDPLQPEL to HLA-B*08:01 is similar to the reported for the binding with a Barr virus peptide, which presented the same pattern and comparable values [57]. According to the RMSF analysis, the binding of the promising peptide increases the flexibility of the two alpha helices of HLA-B*08:01 (GLU58-ALA90 and ASP137-ARG181), as well as the region between the beta strands 2–3 (ASP30), 5–8 (GLY104-ALA136) and 9–10 (PRO193-GLU198); and a large portion of the α3-domain (ALA211-PRO276).

According to the aforementioned, the promising epitopes presented in this study may have an impact in the development of new peptide-based vaccines and diagnostic tests tended to cover Colombian and Latin American population, which also presented a good calculated coverage worldwide. However, further analysis is required and these peptides are proposed as candidates to be submitted to in vitro and in vivo tests.

## 5. Conclusions

This in silico study presents promising T-cell epitopes based on structural proteins of SARS-CoV-2 and HLAs highly frequent in the Colombian population. Some of them with estimated coverage greater than 80%. These peptides were predicted to exhibit immunogenic response, non-allergenicity and non-toxicity. Therefore, these may be useful in the processes of epitope-based vaccine design and diagnostic test development, and are suggested as molecules to be prioritized for further in vitro and in vivo analysis.

## Figures and Tables

**Table 1:** Search queries used to identify allelic frequencies of HLA I and HLA II in the Colombian population on PubMed, Web of Science, and Science Direct (date consulted: 24 February 2021).

Search	Query
HLA Class I	(“MHC Class I” OR “MHC I” OR “HLA Class I” OR “HLA I” OR “HLA-A” OR “HLA-B” OR “HLA-C”) AND “Colombia”
HLA Class II	Query #1: (“MHC Class II” OR “MHC II” OR “HLA Class II” OR “HLA II”) AND “Colombia” Query #2: (“DRB1” OR “DRB3” OR “DRB4” OR “DRB5” OR “DQB1” OR “DQA1”) AND “HLA” AND “Colombia” Query #3: (“DPA1” OR “DPB1”) AND “HLA” AND “Colombia”

**Table 2:** Sequences of the structural proteins of SARS-CoV-2 used for the analysis.

Protein	Length	NCBI Reference Sequence
Spike protein (S)	1273 aa	YP_009724390.1
Envelope protein (E)	75 aa	YP_009724392.1
Membrane glycoprotein (M)	222 aa	YP_009724393.1
Nucleocapsid phosphoprotein (N)	419 aa	YP_009724397.2

**Table 3:** Results retrieved through systematic search on PubMed, Web of Science and Science Direct of HLA I allelic frequencies in the Colombian population (date consulted: 24 February 2021).

HLA I	Results
PubMed	78
Web of Science	116
Science Direct	292

**Table 4:** Results retrieved through systematic search on PubMed, Web of Science and Science Direct of HLA II allelic frequencies in the Colombian population (date consulted: 24 February 2021).

HLA II	Results of Query #1	Results of Query #2	Results of Query #3
PubMed	77	105	6
Web of Science	106	106	6
Science Direct	345	266	40

**Table 5:** Top 10 most frequent HLA-A, HLA-B and HLA-C alleles in the general population of Colombia according to data obtained from the Allele frequency net database (AFND) [28] (date consulted: 24 February 2021). Abbreviations: *n*, sample size.

Ethnic Groups	HLA-A		HLA-B		HLA-C	
	Allele	Frequency (%)	Allele	Frequency (%)	Allele	Frequency (%)
Colombia-Bogotá (*n* = 1463)	A*24:02	20.8	B*35:43	8.6	C*04:01	14.9
	A*02:01	16.1	B*40:02	8.4	C*01:02	11.4
	A*01:01	6.1	B*44:03	5.6	C*07:02	9.7
	A*03:01	6.1	B*51:01	5.6	C*07:01	8.9
	A*68:01	5.2	B*07:02	5.0	C*03:04	8.2
	A*29:02	4.5	B*35:01	4.5	C*05:01	5.1
	A*11:01	4.3	B*14:02	4.0	C*06:02	5.1
	A*31:01	4.0	B*44:02	3.9	C*16:01	5.1
	A*23:01	3.3	B*18:01	3.5	C*08:02	4.9
	A*02:22	3.2	B*08:01	3.2	C*12:03	4.5

**Table 6:** HLA-A, HLA-B and HLA-C alleles with the highest frequency in each Colombian Amerindian groups reported in the Allele frequency net database (AFND) [28] (date consulted: 24 February 2021). Abbreviations: Abbreviations: *n*, sample size; PDB, Protein Data Bank Identifier.

Ethnic Groups	HLA-A		HLA-B		HLA-C	
	Allele	Frequency	Allele	Frequency	Allele	Frequency
Arhuaco (*n* = 17)	A*24:02	0.441	B*35:43	0.441	C*01:02	0.382
Embera (*n* = 14)	A*24:02	0.536	B*39:05	0.429	C*07:02	0.464
Inga (*n* = 16)	A*24:02	0.367	B*40:02	0.286	C*01:02	0.367
Kogi (*n* = 15)	A*24:02	0.433	B*35:43	0.429	C*01:02	0.571
North Chimila (*n* = 47)	A*24:02	0.457	B*51:10	0.404	C*15:02	0.468
North Wiwa El Encanto (*n* = 52)	A*24:02	0.433	B*35:43	0.385	C*01:02	0.51
Waunana (*n* = 20)	A*24:02	0.6	B*40:02	0.25	C*03:04	0.35
Wayuu (*n* = 15)	A*24:02	0.2	B*40:02	0.2	C*04:01	0.25
Zenu (*n* = 16)	A*24:02	0.417	B*40:02	0.25	C*15:02	0.25
Ticuna Arara (*n* = 17)	A*24:02	0.5	B*39:03	0.286	C*07:02	0.429
Ticuna Tarapaca (*n* = 19)	A*24:02	0.526	B*40:02	0.447	C*03:04	0.529

**Table 7:** HLA II alleles with high frequency (WAFs > 5%) in each of the three ethnicities included in this study for the Colombian population.

HLA II Alleles	WAFs (%)	*n*
Mestizo		
DQB1*03:02	22.47	1737
DQB1*03:01	19.23	1737
DQB1*02:01	14.45	1737
DRB1*04:07	12.39	1737
DQB1*05:01	11.63	1737
DQB1*04:02	10.76	1737
DRB1*07:01	9.29	1925
DQB1*06:02	7.03	1737
DRB1*15:01	6.28	1925
DRB1*08:02	6.25	1737
DRB1*03:01	5.74	1925
DRB1*13:01	5.09	1925
African Colombian		
DQA1*01:02	23.42	234
DQA1*05:01	19.86	140
DQB1*02:01	19.79	182
DQA1*01:01	18.04	234
DQB1*05:01	17.69	182
DQB1*06:02	16.21	182
DQB1*03:01	15.88	182
DRB1*15:03	13.47	182
DQA1*03:01	12.77	94
DQB1*04:02	12.37	182
DQA1*04:01	11.29	140
DRB1*03:01	10.98	182
DRB1*03:02	9.17	182
DRB1*07:01	8.56	182
DRB1*08:01	8.30	42
DQA1*02:01	8.04	234
DRB1*08:04	6.00	42
DRB1*13:04	6.00	42
DQB1*03:02	5.94	182
DRB1*13:02	5.65	182
DRB1*01:01	5.14	140
Colombian Amerindians		
DPB1*04:02	49.99	668
DQA1*03:01	46.27	1573
DPB1*14:01	45.67	668
DRB4*01:00	44.23	1300
DQB1*03:02	43.49	2633
DRB4*01:01	38.10	34
DQA1*05:01	35.05	2084
DRB1*04.03	32.30	48
DQB1*03:01	32.06	2633
DQA1*05:00	19.62	321
DQB1*04:02	18.53	2537
DRB5*01:00	18.20	77
DRB3*01:01	18.11	1573
DRB1*14:02	18.01	2173
DQA1*04:01	17.59	2348
DRB1*04:07	17.32	2829
DRB5*02:00	17.11	1257
DRB1*16:02	14.48	2777
DRB1*08:022	14.29	42
DRB1*04:11	14.19	2691
DRB1*08:02	10.47	2701
DRB1*08:04	7.32	2091
DRB1*04:04	7.23	2722

**Table 8:** HLA I and HLA II alleles used in this study for T-cell epitope prediction.

Type	HLA I and HLA II Alleles
HLA-A	HLA-A*24:02, HLA-A*02:01, HLA-A*01:01, HLA-A*03:01, HLA-A*68:01, HLA-A*29:02, HLA-A*11:01, HLA-A*31:01, HLA-A*23:01, and HLA-A*02:22.
HLA-B	HLA-B*35:43, HLA-B*40:02, HLA-B*44:03, HLA-B*51:01, HLA-B*07:02, HLA-B*35:01, HLA-B*14:02, HLA-B*44:02, HLA-B*18:01, HLA-B*08:01, HLA-B*39:05, HLA-B*51:10, and HLA-B*39:03.
HLA-C	HLA-C*04:01, HLA-C*01:02, HLA-C*07:02, HLA-C*07:01, HLA-C*03:04, HLA-C*05:01, HLA-C*06:02, HLA-C*16:01, HLA-C*08:02, HLA-C*12:03, and HLA-C*15:02.
HLA-DRB1	DRB1*01:01, DRB1*03:01, DRB1*03:02, DRB1*04:03, DRB1*04:04, DRB1*04:07, DRB1*04:11, DRB1*07:01, DRB1*08:01, DRB1*08:02, DRB1*08:04, DRB1*08:22, DRB1*13:01, DRB1*13:02, DRB1*13:04, DRB1*14:02, DRB1*15:01, DRB1*15:03, and DRB1*16:02.

**Table 9:** Promising peptides for epitope-based vaccine design from structural proteins of SARS-CoV-2 showing the estimated coverage and the availability of experiments in the immune epitope database (IEDB). These molecules presented predicted immunogenicity, non-toxicity and non-allergenicity. (IEDB, date consulted: 31 March 2021). Abbreviations: ML: MHC ligands assays. TC-IFNg: Tcell assays (IFNg release), TC-A: Tcell assay (Activation), TC-QB: T cell assay (qualitative binding), TC-IL5: T-cell, TC-TNF: T Cell Assays (TNF release), (+): positive, (−): negative. IEDB: Immune Epitope Database, WAF: weighted allelic frequencies.

Epitopes	Estimated Coverage for Colombian Population (WAF)	Experiments (IEDB)	IEDB ID
**Spike Protein (S)**			
**HLA 1**			
VYDPLQPEL	HLA-A = 50.85%, HLA-B = 34.39%, HLA-C = 82.07%.	ML: (+). TC-IFNg: (−).	71996
YQPYRVVVL	HLA-A = 43.43%, HLA-B = 18.49%, HLA-C = 82.07%.	TC-QB: (+).	1334394
TLDSKTQSL	HLA-A = 25.42%, HLA-B = 15.04%, HLA-C = 82.07%.	TC-A: (+). TC-QB: (+). TC-IFNg: (−).	1075075
VRDPQTLEI	HLA-A = 24.09%, HLA-B = 7.42%, HLA-C = 57.39%.		-
FTISVTTEI	HLA-A = 19.34%, HLA-B = 14.84%, HLA-C = 57.5%.	TC-A: (+).	1317060
**HLA 2**			
RAAEIRASANLAATK	HLA-DRB1 = 48.88%.	ML: (+). TC-IFNg: (−).	533447
TPINLVRDLPQGFSA	HLA-DRB1 = 33.7%.	ML: (+).	1330624
FGGFNFSQILPDPSK	HLA-DRB1 = 33.47%.		-
KHTPINLVRDLPQGF	HLA-DRB1 = 31.59%.	TC-A: (+). TC-IFNg: (+). TC-IL5: (−).	1309123
**Envelope Protein (E)**			
**HLA 1**			
YVYSRVKNL	HLA-A = 19.34%, HLA-B = 27.01%, HLA-C = 82.07%.	TC-A: (+). TC-QB: (+).	1075128
LAILTALRL	HLA-B = 6.19%, HLA-C = 22.09%.		-
VSLVKPSFY	HLA-A = 14.9%, HLA-B = 8.65%, HLA-C = 27.97%.		-
VTLAILTAL	HLA-A = 16.13%, HLA-C = 33.47%.		-
RVKNLNSSR	HLA-A = 19.54%.		-
**HLA 2**			
KPSFYVYSRVKNLNS	HLA-DRB1 = 43.18%.		-
VYSRVKNLNSSRVPD	HLA-DRB1 = 55.99%.		-
VKPSFYVYSRVKNLN	HLA-DRB1 = 43.18%.		-
YSRVKNLNSSRVPDL	HLA-DRB1 = 37.1%.		-
SFYVYSRVKNLNSSR	HLA-DRB1 = 61.92%.		-
PSFYVYSRVKNLNSS	HLA-DRB1 = 55.74%.		-
FYVYSRVKNLNSSRV	HLA-DRB1 = 42.58%.	TC-IFNg: (+). TC-TNF: (+). TC-IL5: (−). TC-A: (−).	1310430
LVKPSFYVYSRVKNL	HLA-DRB1 = 26.5%.		-
YVYSRVKNLNSSRVP	HLA-DRB1 = 51.81%.		-
**Membrane Protein (M)**			
HLA 1			
ITVATSRTL	HLA-C = 47.52%.		-
**Nucleocapsid Protein (N)**			
**HLA 1**			
QFAPSASAF	HLA-A = 49.12%, HLA-B = 25.14%, HLA-C = 47.85%.		-
QRNAPRITF	HLA-A = 47.09%, HLA-B = 19.77%, HLA-C = 24.33%.	TC-IFNg: (+). TC-QB: (+). TC-TNF: (−). TC-A: (−).	1309136
SPDDQIGYY	HLA-A = 3.24%, HLA-B = 25.38%, HLA-C = 26%.	TC-A: (+). TC-IFNg: (−).	1310816
**HLA 2**			
GTWLTYTGAIKLDDK	HLA-DRB1 = 40.37%.	TC-IFNg: (+). TC-A: (+). TC-TNF: (+).	1310464
NFKDQVILLNKHIDA	HLA-DRB1 = 24.69%.		-
TKAYNVTQAFGRRGP	HLA-DRB1 = 52.85%.		-

**Table 10:** Promising peptides predicted to induce the release of TNF gamma showing the worldwide estimated coverage calculated by IEDB. ND: no data available.

Epitopes	SARS-CoV-2 Protein	IFN Epitope Server	Worldwide Coverage (%) [IEDB]
YQPYRVVVL	S	0.29285355	96.62
RAAEIRASANLAATK	S	0.29346657	ND
QFAPSASAF	N	0.82212984	77.60
SPDDQIGYY	N	0.24883001	80.12

## Data Availability

Data is contained within the article.

## Supplementary Materials

The following are available online at https://www.mdpi.com/article/10.3390/vaccines9070797/s1: Python Scripts: Analysis_HLA_I.py, Analysis_HLA_II.py, Interactions_Summary.py, Python_Script_CumFreq.py, and Python_Script_WAF.py. Figure S1: Three-dimensional view of the overall structures (left) and predicted peptide-protein interactions (right) of the complexes formed by HLA-A*24:02 with the SARS-CoV-2 promising peptides: (A) YQPYRVVVL, (B) RAAEIRASANLAATK, (C) SPDDQIGYY, and (D) QFAPSASAF. Figure S2: Three-dimensional view of the overall structures (left) and predicted peptide-protein interactions (right) of the complexes formed by HLA-B*51:04 with the SARS-CoV-2 promising peptides: (A) YQPYRVVVL, (B) RAAEIRASANLAATK, (C) SPDDQIGYY, and (D) QFAPSASAF. Figure S3: Three-dimensional view of the overall structures (left) and predicted peptide-protein interactions (right) of the complexes formed by HLA-C*04:01 with the SARS-CoV-2 promising peptides: (A) YQPYRVVVL, (B) RAAEIRASANLAATK, (C) SPDDQIGYY, and (D) QFAPSASAF. Figure S4: Three-dimensional view of the overall structures (left) and predicted peptide-protein interactions (right) of the complexes formed by HLA-DQA1*01:02 (gray)/HLA-DQB1*06:02 (green; PDB ID: 6DIG) with the SARS-CoV-2 promising peptides: A) YQPYRVVVL, B) RAAEIRASANLAATK, C) SPDDQIGYY, and D) QFAPSASAF. Table S1. HLAs commonly found in the Colombian population with three-dimensional structures available in Protein Data Bank (PDB) used in this study. Table S2. Results of the systematic search for HLA I commonly present in the Colombian population. Table S3. Results of the systematic search for HLA II commonly present in the Colombian population. Table S4. Weighted allelic frequencies (WAFs) of HLA II in the Colombian population calculated as the weighted average of the frequencies obtained from the literature search and AFND. Table S5. HLA I epitopes generated by NetMHCpan 4.1 based on the S protein of SARS-CoV-2. Table S6. HLA I epitopes generated by NetMHCpan 4.1 based on the N protein of SARS-CoV-2. Table S7. HLA I epitopes generated by NetMHCpan 4.1 based on the E protein of SARS-CoV-2. Table S8. HLA I epitopes generated by NetMHCpan 4.1 based on the M protein of SARS-CoV-2. Table S9. HLA II epitopes generated by NetMHCIIpan 4.0 based on the S protein of SARS-CoV-2. Table S10. HLA II epitopes generated by NetMHCIIpan 4.0 based on the N protein of SARS-CoV-2. Table S11. HLA II epitopes generated by NetMHCIIpan 4.0 based on the E protein of SARS-CoV-2. Table S12. HLA II epitopes generated by NetMHCIIpan 4.0 based on the M protein of SARS-CoV-2. Table S13. AutoDock Vina docking affinity scores (kcal/mol) predicted for the interaction between the promising peptides and HLAs commonly found in the Colombian population.

## References

[1] Galanakis C.M. (2020). The food systems in the era of the Coronavirus (COVID-19) pandemic crisis. Foods.

[2] World Health Organization Weekly Epidemiological Update on COVID-19—6 July 2021. https://www.who.int/publications/m/item/weekly-epidemiological-update-on-covid-19---6-july-2021.

[3] World Health Organization WHO Coronavirus (COVID-19) Dashboard|WHO Coronavirus Disease (COVID-19) Dashboard. https://covid19.who.int/.

[4] Acter T., Uddin N., Das J., Akhter A., Choudhury T.R., Kim S. (2020). Evolution of severe acute respiratory syndrome coronavirus 2 (SARS-CoV-2) as coronavirus disease 2019 (COVID-19) pandemic: A global health emergency. Sci. Total Environ..

[5] Al-Rohaimi A.H., Al Otaibi F. (2020). Novel SARS-CoV-2 outbreak and COVID19 disease; a systemic review on the global pandemic. Genes Dis..

[6] Bahrami M., Kamalinejad M., Latifi S.A., Seif F., Dadmehr M. (2020). Cytokine storm inCOVID-19 and parthenolide: Preclinical evidence. Phyther. Res..

[7] Karwaciak I., Sałkowska A., Karaś K., Dastych J., Ratajewski M. (2021). Nucleocapsid and spike proteins of the Coronavirus SARS-CoV-2 induce IL6 in monocytes and macrophages—Potential implications for cytokine storm syndrome. Vaccines.

[8] Bachmann M.F., Mohsen M.O., Zha L., Vogel M., Speiser D.E. (2021). SARS-CoV-2 structural features may explain limited neutralizing-antibody responses. NPJ Vaccines.

[9] Veldhoen M., Simas J.P. (2021). Endemic SARS-CoV-2 will maintain post-pandemic immunity. Nat. Rev. Immunol..

[10] Rastogi M., Pandey N., Shukla A., Singh S.K. (2020). SARS coronavirus 2: From genome to infectome. Respir. Res..

[11] Pooladanda V., Thatikonda S., Godugu C. (2020). The current understanding and potential therapeutic options to combat COVID-19. Life Sci..

[12] Vellingiri B., Jayaramayya K., Iyer M., Narayanasamy A., Govindasamy V., Giridharan B., Ganesan S., Venugopal A., Venkatesan D., Ganesan H. (2020). COVID-19: A promising cure for the global panic. Sci. Total Environ..

[13] Tahirul Qamar M., Alqahtani S.M., Alamri M.A., Chen L.L. (2020). Structural basis of SARS-CoV-2 3CLpro and anti-COVID-19 drug discovery from medicinal plants. J. Pharm. Anal..

[14] Satarker S., Nampoothiri M. (2020). Structural proteins in severe acute respiratory syndrome Coronavirus-2. Arch. Med. Res..

[15] Mandala V.S., McKay M.J., Shcherbakov A.A., Dregni A.J., Kolocouris A., Hong M. (2020). Structure and drug binding of the SARS-CoV-2 envelope protein transmembrane domain in lipid bilayers. Nat. Struct. Mol. Biol..

[16] Lu S., Ye Q., Singh D., Cao Y., Diedrich J.K., Yates J.R., Villa E., Cleveland D.W., Corbett K.D. (2021). The SARS-CoV-2 nucleocapsid phosphoprotein forms mutually exclusive condensates with RNA and the membrane-associated M protein. Nat. Commun..

[17] Thomas S. (2020). The structure of the membrane protein of sars-cov-2 resembles the sugar transporter semisweet. Pathog. Immun..

[18] Ashik A.I., Hasan M., Tasnim A.T., Chowdhury M.B., Hossain T., Ahmed S. (2020). An immunoinformatics study on the spike protein of SARS-CoV-2 revealing potential epitopes as vaccine candidates. Heliyon.

[19] Sohail M.S., Ahmed S.F., Quadeer A.A., McKay M.R. (2021). In silico T cell epitope identification for SARS-CoV-2: Progress and perspectives. Adv. Drug Deliv. Rev..

[20] Ita K. (2021). Coronavirus disease (COVID-19): Current status and prospects for drug and vaccine development. Arch. Med. Res..

[21] Requena D., Médico A., Chacón R.D., Ramírez M., Marín-Sánchez O. (2020). Identification of novel candidate epitopes on SARS-CoV-2 proteins for South America: A review of HLA frequencies by country. Front. Immunol..

[22] Tan H.-X., Juno J.A., Lee W.S., Barber-Axthelm I., Kelly H.G., Wragg K.M., Esterbauer R., Amarasena T., Mordant F.L., Subbarao K. (2021). Immunogenicity of prime-boost protein subunit vaccine strategies against SARS-CoV-2 in mice and macaques. Nat. Commun..

[23] Dobaño C., Santano R., Jiménez A., Vidal M., Chi J., Melero N.R., Popovic M., López-Aladid R., Fernández-Barat L., Tortajada M. (2021). Immunogenicity and crossreactivity of antibodies to the nucleocapsid protein of SARS-CoV-2: Utility and limitations in seroprevalence and immunity studies. Transl. Res..

[24] Diao B., Wen K., Zhang J., Chen J., Han C., Chen Y., Wang S., Deng G., Zhou H., Wu Y. (2021). Accuracy of a nucleocapsid protein antigen rapid test in the diagnosis of SARS-CoV-2 infection. Clin. Microbiol. Infect..

[25] Peng Y., Mentzer A.J., Liu G., Yao X., Yin Z., Dong D., Dejnirattisai W., Rostron T., Supasa P., Liu C. (2020). Broad and strong memory CD4+ and CD8+ T cells induced by SARS-CoV-2 in UK convalescent individuals following COVID-19. Nat. Immunol..

[26] Nelde A., Bilich T., Heitmann J.S., Maringer Y., Salih H.R., Roerden M., Lübke M., Bauer J., Rieth J., Wacker M. (2021). SARS-CoV-2-derived peptides define heterologous and COVID-19-induced T cell recognition. Nat. Immunol..

[27] Kiyotani K., Toyoshima Y., Nemoto K., Nakamura Y. (2020). Bioinformatic prediction of potential T cell epitopes for SARS-Cov-2. J. Hum. Genet..

[28] Gonzalez-Galarza F.F., McCabe A., dos Santos E.J.M., Jones J., Takeshita L., Ortega-Rivera N.D., Cid-Pavon G.M.D., Ramsbottom K., Ghattaoraya G., Alfirevic A. (2020). Allele frequency net database (AFND) 2020 update: Gold-standard data classification, open access genotype data and new query tools. Nucleic Acids Res..

[29] Arnaiz-Villena A., Palacio-Gruber J., Juarez I., Hernandez E., Muniz E., Bayona B., Campos C., Nieto J., Martin-Villa M., Silvera C. (2018). HLA in North Colombia Chimila Amerindians. Hum. Immunol..

[30] Arnaiz-Villena A., Palacio-Gruber J., Juarez I., Muniz E., Hernandez E., Bayona B., Campos C., Nunez J., Lopez-Nares A., Martin-Villa M. (2018). Study of Colombia North Wiwa el encanto Amerindians HLA- genes: Pacific Islanders relatedness. Hum. Immunol..

[31] Single R.M., Meyer D., Nunes K., Francisco R.S., Hunemeier T., Maiers M., Hurley C.K., Bedoya G., Gallo C., Hurtado A.M. (2020). Demographic history and selection at HLA loci in Native Americans. PLoS ONE.

[32] Páez-Gutiérrez I.A., Hernández-Mejía D.G., Vanegas D., Camacho-Rodríguez B., Perdomo-Arciniegas A.M. (2019). HLA-A, -B, -C, -DRB1 and -DQB1 allele and haplotype frequencies of 1463 umbilical cord blood units typed in high resolution from Bogotá, Colombia. Hum. Immunol..

[33] Reynisson B., Alvarez B., Paul S., Peters B., Nielsen M. (2021). NetMHCpan-4.1 and NetMHCIIpan-4.0: Improved predictions of MHC antigen presentation by concurrent motif deconvolution and integration of MS MHC eluted ligand data. Nucleic Acids Res..

[34] Doytchinova I.A., Flower D.R. (2007). VaxiJen: A server for prediction of protective antigens, tumour antigens and subunit vaccines. BMC Bioinform..

[35] Dimitrov I., Flower D.R., Doytchinova I. (2013). AllerTOP—A server for in silico prediction of allergens. BMC Bioinform..

[36] Gupta S., Kapoor P., Chaudhary K., Gautam A., Kumar R., Raghava G.P.S. (2013). In silico approach for predicting toxicity of peptides and proteins. PLoS ONE.

[37] Thévenet P., Shen Y., Maupetit J., Guyon F., Derreumaux P., Tufféry P. (2012). PEP-FOLD: An updated de novo structure prediction server for both linear and disulfide bonded cyclic peptides. Nucleic Acids Res..

[38] Burley S.K., Berman H.M., Bhikadiya C., Bi C., Chen L., di Costanzo L., Christie C., Dalenberg K., Duarte J.M., Dutta S. (2019). RCSB Protein Data Bank: Biological macromolecular structures enabling research and education in fundamental biology, biomedicine, biotechnology and energy. Nucleic Acids Res..

[39] Morris G.M., Huey R., Lindstrom W., Sanner M.F., Belew R.K., Goodsell D.S., Olson A.J. (2009). Autodock4 and AutoDockTools4: Automated docking with selective receptor flexiblity. J. Comput. Chem..

[40] Trott O., Olson A.J. (2010). AutoDock Vina: Improving the speed and accuracy of docking with a new scoring function, efficient optimization, and multithreading. J. Comput. Chem..

[41] (2019). R Core Development Team R.

[42] Warnes G.R., Bolker B., Bonebakker L., Gentleman R., Huber W., Liaw A., Lumley T., Maechler M., Magnusson A., Moeller S. (2009). Gplots: Various R programming tools for plotting data. R Packag..

[43] Laskowski R.A., Swindells M.B. (2011). LigPlot+: Multiple ligand-protein interaction diagrams for drug discovery. J. Chem. Inf. Model..

[44] Abraham M.J., Murtola T., Schulz R., Páll S., Smith J.C., Hess B., Lindah E. (2015). Gromacs: High performance molecular simulations through multi-level parallelism from laptops to supercomputers. SoftwareX.

[45] MacKerell A.D., Bashford D., Bellott M., Dunbrack R.L., Evanseck J.D., Field M.J., Fischer S., Gao J., Guo H., Ha S. (1998). All-atom empirical potential for molecular modeling and dynamics studies of proteins. J. Phys. Chem. B.

[46] Painter C.A., Cruz A., López G.E., Stern L.J., Zavala-Ruiz Z. (2008). Model for the peptide-free conformation of class II MHC proteins. PLoS ONE.

[47] Mukherjee S., Tworowski D., Detroja R., Mukherjee S.B., Frenkel-Morgenstern M. (2020). Immunoinformatics and structural analysis for identification of immunodominant epitopes in SARS-CoV-2 as potential vaccine targets. Vaccines.

[48] Smith K.D., Kurago Z.B., Lutz C.T. (1997). Conformational changes in MHC class I molecules. Antibody, T-cell receptor, and NK cell recognition in an HLA-B7 model system. Immunol. Res..

[49] Chen Z., Ruan P., Wang L., Nie X., Ma X., Tan Y. (2021). T and B cell Epitope analysis of SARS-CoV-2 S protein based on immunoinformatics and experimental research. J. Cell. Mol. Med..

[50] Chen H.-Z., Tang L.-L., Yu X.-L., Zhou J., Chang Y.-F., Wu X. (2020). Bioinformatics analysis of epitope-based vaccine design against the novel SARS-CoV-2. Infect. Dis. Poverty.

[51] World Health Organization Tracking SARS-CoV-2 Variants. https://www.who.int/en/activities/tracking-SARS-CoV-2-variants/.

[52] Poran A., Harjanto D., Malloy M., Arieta C.M., Rothenberg D.A., Lenkala D., van Buuren M.M., Addona T.A., Rooney M.S., Srinivasan L. (2020). Sequence-based prediction of SARS-CoV-2 vaccine targets using a mass spectrometry-based bioinformatics predictor identifies immunogenic T cell epitopes. Genome Med..

[53] Ambrose J.M., Veeraraghavan V.P., Kullappan M., Chellapandiyan P., Mohan S.K., Manivel V.A. (2021). Comparison of immunological profiles of SARS-CoV-2 variants in the COVID-19 pandemic trends: An immunoinformatics approach. Antibiotics.

[54] Anand R., Biswal S., Bhatt R., Tiwary B.N. (2020). Computational perspectives revealed prospective vaccine candidates from five structural proteins of novel SARS corona virus 2019 (SARS-CoV-2). PeerJ.

[55] Lim H.X., Lim J., Jazayeri S.D., Poppema S., Poh C.L. (2021). Development of multi-epitope peptide-based vaccines against SARS-CoV-2. Biomed. J..

[56] Van Deutekom H.W.M., Keşmir C. (2015). Zooming into the binding groove of HLA molecules: Which positions and which substitutions change peptide binding most?. Immunogenetics.

[57] Knapp B., Deane C.M. (2015). T-cell receptor binding affects the dynamics of the peptide/MHC-I complex. J. Chem. Inf. Model..

