# Supplementary material for: Bioinformatics Prediction of SARS-CoV-2 Epitopes as Vaccine Candidates for the Colombian Population
Source: Vaccines (Basel). 2021 Jul 17;9(7):797. doi: 10.3390/vaccines9070797 (PMC8310250; doi:10.3390/vaccines9070797)
Supplement: Supplementary file 1 [file vaccines-09-00797-s001.zip › Table_S1.pdf]

SUPPLEMENTARY MATERIAL

HLAs	PDB ID
**HLA I**	
A*01:01	4NQX
A*03:01	6O9B
A*11:01	1X7Q
A*24:02	2BCK
A*68:01	6PBH
B*07:02	5EO0
B*08:01	3X13
B*14:02	3BVN
B*18:01	6MT3
B*35:01	1XH3
B*44:02	1M6O
B*44:03	4JQX
B*51:01	5VUE
C*03:04	1EFX
C*04:01	1QQD
C*05:01	5VGD
C*06:02	5W67
C*07:02	5VGE
**HLA II**	
DQA1*01:02/DQB1*06:02	6DIG
DQA1*03:01/DQB1*02:01	4D8P
DRB1*01:01	5V4N
DRB1*1402	6ATF
DRB1*15:01	5V4M
DRB3*01:01	2Q6W

Table S1. HLAs commonly found in Colombian population with three-dimensional structures available in Protein Data Bank (PDB) used in this study.
