# Supplementary figures and images for: Bioinformatics Prediction of SARS-CoV-2 Epitopes as Vaccine Candidates for the Colombian Population
Source: Vaccines (Basel). 2021 Jul 17;9(7):797. doi: 10.3390/vaccines9070797 (PMC8310250; doi:10.3390/vaccines9070797)

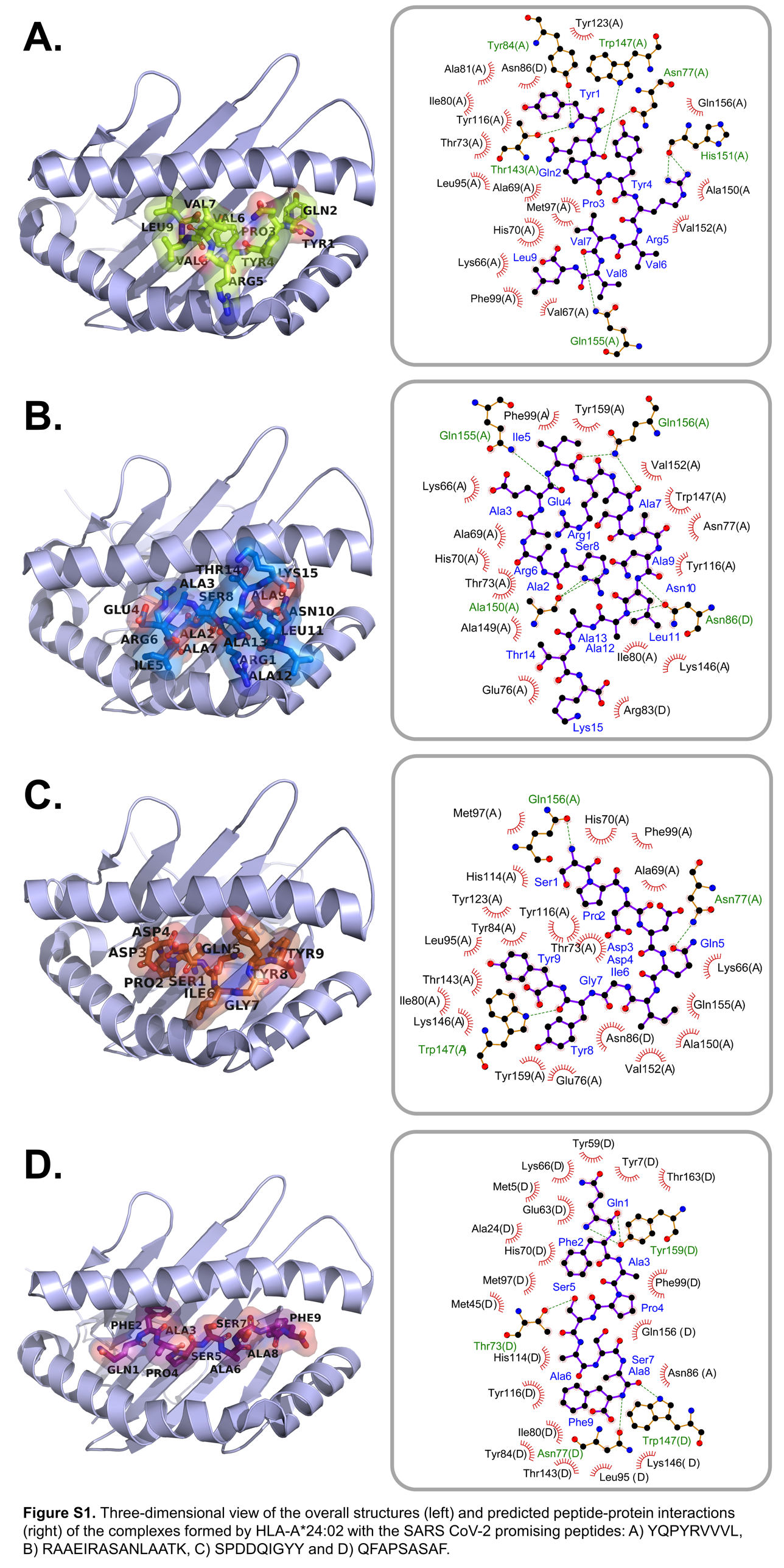

Supplement: Supplementary file 1 [file vaccines-09-00797-s001.zip › Figure_S1_2bck_FV.tif]

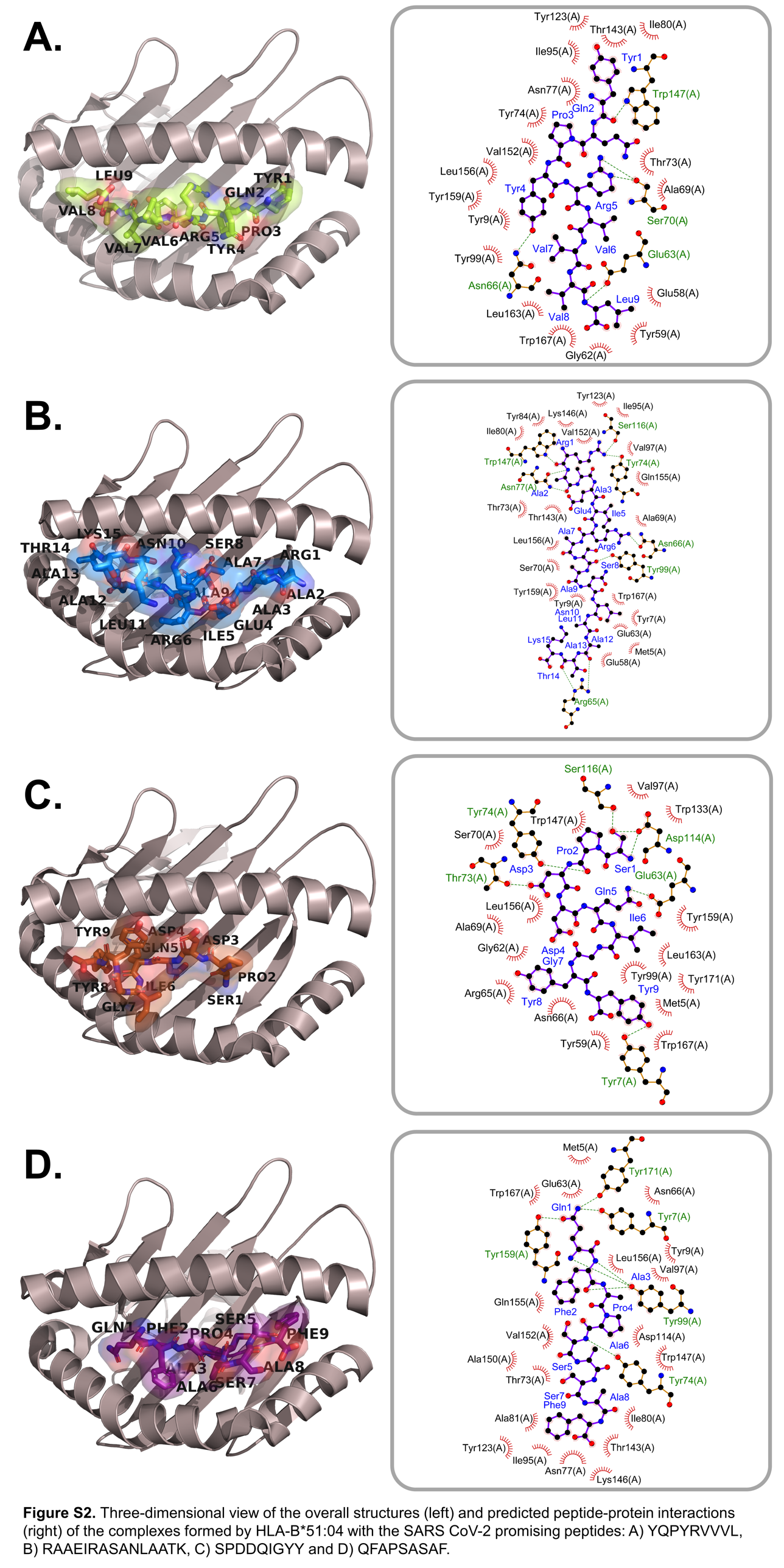

Supplement: Supplementary file 1 [file vaccines-09-00797-s001.zip › Figure_S2_5vue_FV.tif]

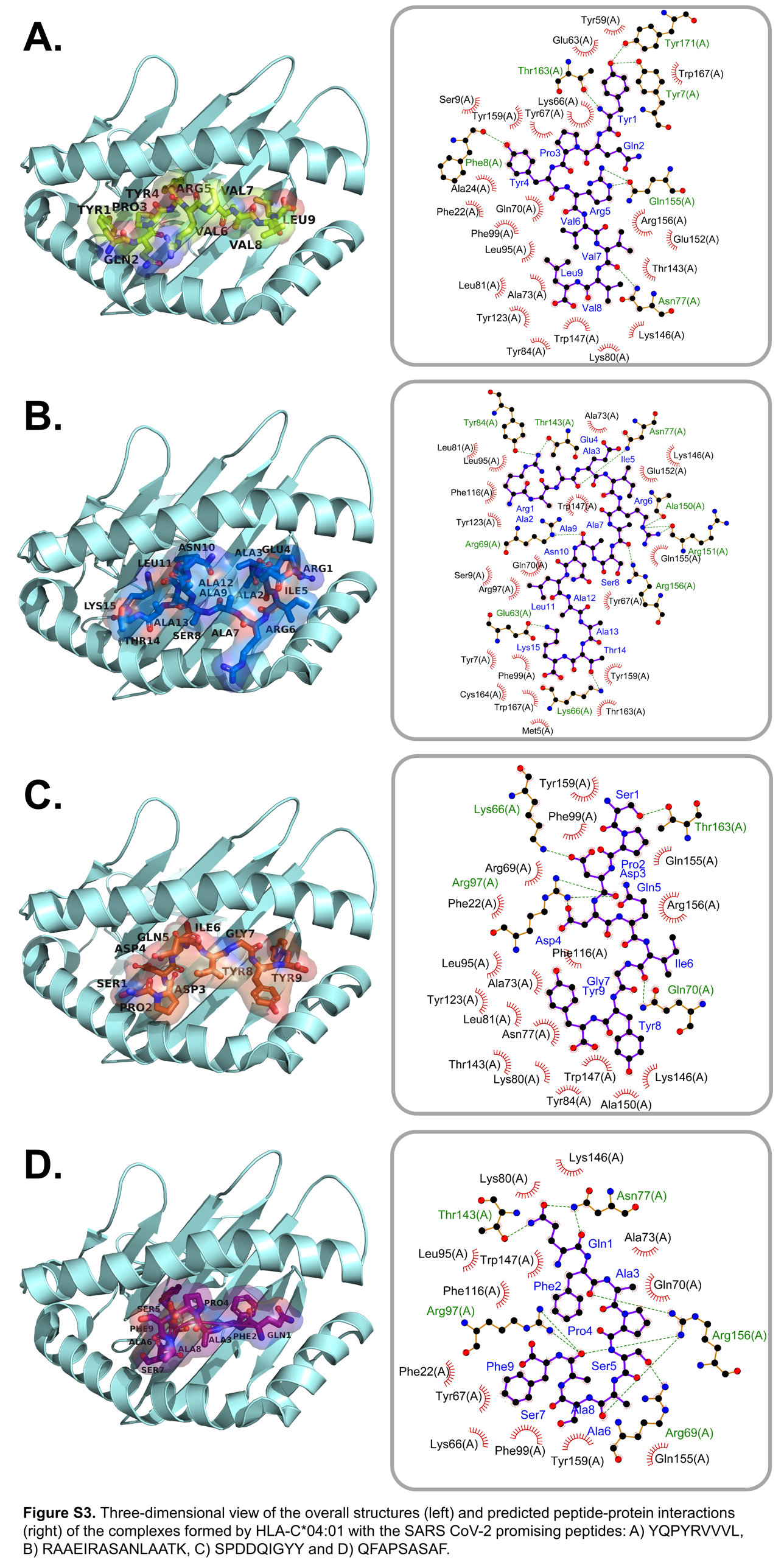

Supplement: Supplementary file 1 [file vaccines-09-00797-s001.zip › Figure_S3_1qqd_FV.tif]

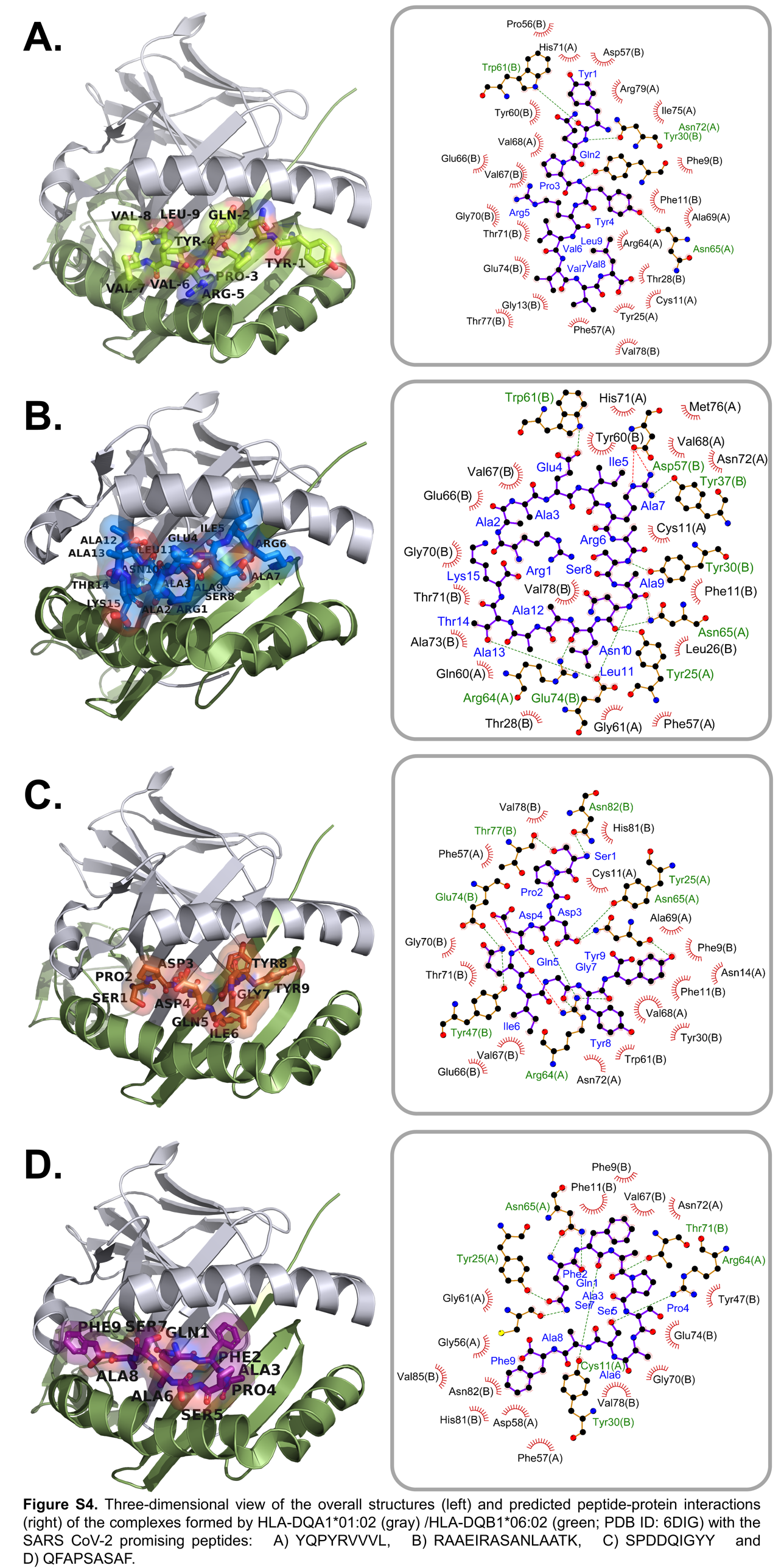

Supplement: Supplementary file 1 [file vaccines-09-00797-s001.zip › Figure_S4_6dig_FV.tif]
